# Catatonic Schizophrenia: Cases with Possible Genetic Predisposition

**DOI:** 10.7759/cureus.4525

**Published:** 2019-04-23

**Authors:** Maryam Tariq, Muhammad Iqbal Afridi, Dua Saleem, Sarmad Pirzada

**Affiliations:** 1 Psychiatry and Behavioral Sciences, Jinnah Postgraduate Medical Center, Karachi, PAK; 2 Psychiatry and Behavioural Sciences, Jinnah Postgraduate Medical Center, Karachi, PAK; 3 Internal Medicine, Dow University of Health Sciences, Karachi, PAK

**Keywords:** catatonic schizophrenia, catatonia, genetic, hereditary, schizophrenia, olanzapine, lorazepam, aripiprazole, brothers, mutism

## Abstract

Catatonic schizophrenia is defined by catatonia seen either with alternating phases of stupor and motor rigidity or the extreme phase of catatonic excitement. This variant of schizophrenia has been identified with poor prognosis, mainly due to the higher association with negative symptoms and young age onset. In this paper, we illustrate a similar clinical picture of catatonic schizophrenia in two brothers, with no genetic predisposition to schizophrenia and no proximal stressors apart from the aggressive/violent behavior of their elder brother.

Case presentation 1 (Patient A): An 18-year-old male from a lower socio-economic class with no previous mental health issues presented to the emergency department with complete mutism, marked psychomotor retardation, posturing along with a refusal to drink or eat, and complete lack of self-care for about two months. The diagnosis of catatonic schizophrenia was made, and the patient was started orally on aripiprazole and lorazepam. On the third day of admission, noticeable changes were observed, and in the following days, he started eating and going to the toilet while still being completely mute. After two weeks on treatment, he started responding with one-word answers.

Case presentation 2 (Patient B): The biological brother of patient A, a 30-year-old male, presented on the same day with an identical history of mutism, decreased psychomotor activity, posturing along with a refusal to drink or eat, and lack of self-care for the past few months. The diagnosis of catatonic schizophrenia was made. The patient was started orally on both, olanzapine and lorazepam. He showed a quicker response to treatment with the maintenance of eye contact on the second day of treatment and started giving short answers to questions on the fifth post-admission day.

We here discuss a possible genetic predisposition to catatonic schizophrenia and its initial improvement with lorazepam and subsequent treatment with olanzapine proving to be more efficacious than aripiprazole.

## Introduction

Catatonic schizophrenia is defined by catatonia seen either with alternating phases of stupor and motor rigidity, or the extreme phase of catatonic excitement manifesting as echolalia and echopraxia [1]. This phenomenon is seen much more rarely than before, due to advancements in the medical treatments available now [1]. The decline of this catatonic subtype was strongly authenticated by Tempter and Velebor [2].

Some of the most common signs of catatonic schizophrenia are certain mannerisms, grimacing, stereotypy, and mutism [3]. However, the diagnosis remains confined to the clinical assessment rather than being limited to the signs and symptoms mentioned above. Moreover, this variant of schizophrenia has been identified with poor prognosis, mainly due to the higher association with negative symptoms and young age onset [4].

In this paper, we illustrate a similar clinical picture of catatonic schizophrenia in two brothers, with no genetic predisposition to schizophrenia and no proximal stressors apart from the aggressive/violent behavior of their elder brother.

## Case presentation

Case presentation 1

Patient A, an 18-year-old male from a lower socio-economic class with no prior mental health issues presented to the emergency department with complete mutism, marked psychomotor retardation, posturing along with a refusal to drink or eat, and complete lack of self-care for about two months. The onset of the disease was gradual with positive psychotic symptoms of bizarre delusions and auditory hallucinations over a span of 14 months, but then his condition gradually deteriorated over months to marked negative symptoms and catatonia. Self-care decreased to the point of passing urine and stool in the clothes for the past few months. He did not show any signs of echolalia, echopraxia, negativism, waxy flexibility, mitgahen/mitmachen, or ambitendency.

In these approximately one-and-a-half years, the patient’s family consulted faith healers for spiritual treatment, but it did not lead to any improvement in symptoms. Thus, in his first hospital admission, in accordance with the criteria of Statistical Classification of Diseases and Related Health Problems (ICD-10) [5], the confirmative diagnosis of catatonic schizophrenia was made, and the patient was started on aripiprazole (15 mg a day) and lorazepam (1 mg thrice a day). Both aripiprazole and lorazepam were administered orally. There were no signs of improvement on the first two days, but on the third day of admission, noticeable changes were observed, and he started making eye contact. On the following days, he started eating and going to the toilet. The patient was still completely mute. After two weeks on treatment, he started responding with one-word answers. Along with clinical assessment, the response was also measured with the help of psychometric tools, the Positive and Negative Syndrome Scale (PANSS) and the Brief Psychiatric Rating Scale (BPRS).

Case presentation 2

Patient B, the biological brother of Patient A, a 30-year-old male, presented on the same day with an identical history of mutism, decreased psychomotor activity, posturing along with a refusal to drink or eat, and lack of self-care for the past few months. The psychotic symptoms had developed gradually over two years, and there was a similar history of passing urine in the clothes for the last two months. The patient was in a catatonic stupor and did not respond to any commands either verbally or physically. Like his brother, he was also taken to spiritual healers which did not prove to be beneficial. Thus, in his first hospital admission, after a detailed history was taken and mental state and neurological examinations and relevant detailed investigations were made, a formal diagnosis of catatonic schizophrenia was made according to the criteria set by ICD-10. Features of echolalia, echopraxia, negativism, waxy flexibility, mitgahen/mitmachen or ambitendency were not evident.

The patient was started orally on both, olanzapine (10 mg a day) and lorazepam (1 mg twice a day). The response of the patient was measured clinically and the psychometric tools PANSS and BPRS were also used. He showed a quicker response to treatment with the maintenance of eye contact on the second day of treatment and started giving short answers to questions on the fifth post-admission day. Level of self-care was also relatively better than his brother at the time of discharge.

Investigations

Patient A and Patient B were biological brothers. On examination, their vital signs were within normal parameters. Neurological examination was grossly normal in both. To rule out any organic brain disease, hypothyroidism, or Wilson's disease, magnetic resonance imaging (MRI) brain full study, thyroid profile, serum ceruloplasmin levels, 24-hour urinary copper, and slit lamp examination for the detection of Kayser-Fleischer rings were done. All investigations were unremarkable.

Both the patients did not have any history of convulsions, head injury, a high-grade fever, dog bite, history of tuberculosis (TB) or TB contact nor any other features suggesting organicity. There was no history of substance use. The only identified psychosocial stressor was the intra-personal issues within the family. The physically and verbally abusive behavior of their elder brother led to highly expressed emotions in the family and disturbed the overall family dynamics. The pre-morbid functionality of both the patients was satisfactory and there was no apparent stress factor at work.

## Discussion

Here we describe a case of two biological brothers suffering from similar signs and symptoms of catatonic schizophrenia. Historically, catatonia was considered a subtype of schizophrenia, but recent studies have shown it to be a neuropsychiatric syndrome comprising psychomotor inhibition that occurs in greater than 10% of patients suffering from acute psychiatric disorders [6]. The association of catatonia with numerous psychiatric disorders and somatic diseases necessitates a meticulous differential diagnosis based on the focal history, clinical findings, laboratory investigations, and neurological examination. Catatonic symptoms often resemble those of neuroleptic malignant syndrome (NMS). In this case, NMS and drug-induced parkinsonism were ruled out since the signs and symptoms were present before the administration of antipsychotics. The unremarkable laboratory findings of the electroencephalogram (EEG) and magnetic resonance imaging (MRI) in our case eliminated the possibilities of akinetic mutism, nonconvulsive status elipticus, vegetative state, and locked-in syndrome in both the patients. Noncatatonic stupor was also excluded due to the absence of any prior trauma to the head. Therefore, after the exclusion of all other possibilities, the diagnosis of catatonic schizophrenia was made according to the diagnostic criteria of ICD-10.

The existing literature records stereotypy, perseveration, mannerisms, and posturing to be the usual clinical presentation of the chronic type of catatonic schizophrenia. However, the most prominent catatonic signs in our case were mutism, psychomotor retardation, and posturing which are in accordance with a study conducted by Bush et al., in 1997 [7]. Studies by Kallmann [8], and Scharfetter and Nüsperli [9] have established catatonic schizophrenia to have more genetic susceptibility than the other subtypes of schizophrenia. This has also been proven by several subsequent studies. A 1996 family study conducted by Beckman et al. [10] reported periodic catatonia to be a familial disorder, and this was confirmed by Gerald Stöber et al., who discovered chromosome 15q15 to be a host of a major disease locus associated with periodic catatonia [11]. Along with significant evidence on chromosome 15q15, research about catatonic schizophrenia in 12 German multiplex pedigrees also revealed a possible link to chromosome 22q13 [11-12]. The study conducted by Meyer et al. reports a missense mutation of the WKL1 gene on chromosome 22q13 to be a cause for familial catatonic schizophrenia [13]. Our patients were biological brothers both suffering from chronic catatonia with similar etiologies; this supports the fact that there exists a genetic susceptibility to developing chronic catatonia. Although the genes were previously unexpressed in the pedigree, as suggested by their negative family history, we hypothesize the possibility of silent genes being passed on and getting phenotypically expressed with time along with a probable contribution from the shared environmental risk factors.

Much like its definition, the treatment of catatonia has long been subject to debate. Unlike some cases, which report poor response to benzodiazepines [14], lorazepam was responsible for the initial improvement seen in our case. A report by Singh and Praharaj has also recorded lorazepam to be highly efficacious [15]; hence, there is no fixed rule about its administration in catatonic schizophrenia. Some researchers also suggest a difference in the treatment of acute and chronic types of catatonia [4]. However, Gaind GS et al. reported that lorazepam worked efficiently in two mentally retarded brothers, where one suffered from acute catatonia and the other from chronic catatonia [16]. Apart from benzodiazepines, other treatment regimens used for catatonic schizophrenia include antipsychotics, glutamate antagonists, GABA agonists, and electroconvulsive therapy [17]. The Maudsley Prescribing Guidelines in Psychiatry (13th Edition) supports the use of second-generation antipsychotics like aripiprazole and olanzapine for catatonia associated with psychotic symptoms [18]. Our treatment was in accordance with the guidelines and aligns with the group of cases where antipsychotics proved efficacious. Olanzapine administered to our 30-year-old patient has previously been successful in treating chronic catatonia [19]. Correspondingly, Kirino E recorded aripiprazole to be effectual in treating catatonic schizophrenia in an adolescent male [20], and our case of the 18-year-old patient concurs with this result. However, in comparison to aripiprazole, our case demonstrated olanzapine to be quicker in alleviating catatonic symptoms, especially mutism.

## Conclusions

Due to a multitude of factors, reaching a definitive cause for catatonic schizophrenia can be challenging. Here, the case of two biological brothers suffering from similar signs and symptoms of catatonic schizophrenia reveals the possible genetic contribution to the disorder, the catatonic subtype being itself a rare phenomenon. Secondly, our treatment regimen proves olanzapine to have a greater effect in alleviating the symptoms of the disease compared to aripiprazole.

Therefore, we recommend future studies to investigate the etiology of catatonic schizophrenia, its genetic predisposition, and the susceptibility of certain populations to develop this condition.
